# Early Linkage to HIV Care and Antiretroviral Treatment among Men Who Have Sex with Men — 20 Cities, United States, 2008 and 2011

**DOI:** 10.1371/journal.pone.0132962

**Published:** 2015-07-15

**Authors:** Brooke E. Hoots, Teresa J. Finlayson, Cyprian Wejnert, Gabriela Paz-Bailey, NHBS Study Group

**Affiliations:** Division of HIV/AIDS Prevention, National Center for HIV, Viral Hepatitis, STD, and TB Prevention, Centers for Disease Control and Prevention, Atlanta, Georgia, United States of America; Emory University's RSPH, UNITED STATES

## Abstract

Early linkage to care and antiretroviral (ARV) treatment are associated with reduced HIV transmission. Male-to-male sexual contact represents the largest HIV transmission category in the United States; men who have sex with men (MSM) are an important focus of care and treatment efforts. With the release of the National HIV/AIDS Strategy and expanded HIV treatment guidelines, increases in early linkage to care and ARV treatment are expected. We examined differences in prevalence of early linkage to care and ARV treatment among HIV-positive MSM between 2008 and 2011. Data are from the National HIV Behavioral Surveillance System, which monitors behaviors among populations at high risk of HIV infection in 20 U.S. cities with high AIDS burden. MSM were recruited through venue-based, time-space sampling. Prevalence ratios comparing 2011 to 2008 were estimated using linear mixed models. Early linkage was defined as an HIV clinic visit within 3 months of diagnosis. ARV treatment was defined as use at interview. Prevalence of early linkage to care was 79% (187/236) in 2008 and 83% (241/291) in 2011. In multivariable analysis, prevalence of early linkage did not differ significantly between years overall (*P* = 0.44). Prevalence of ARV treatment was 69% (790/1,142) in 2008 and 79% (1,049/1,336) in 2001. In multivariable analysis, ARV treatment increased overall (*P* = 0.0003) and among most sub-groups. Black MSM were less likely than white MSM to report ARV treatment (*P* = 0.01). While early linkage to care did not increase significantly between 2008 and 2011, ARV treatment increased among most sub-groups. Progress is being made in getting MSM on HIV treatment, but more efforts are needed to decrease disparities in ARV coverage.

## Introduction

Men who have sex with men (MSM) are at increased risk of HIV infection and male-to-male sexual contact represents the largest HIV transmission category in the United States. Of the 47,500 estimated incident HIV infections in the United States in 2010, 66% were estimated to be among MSM, including MSM who inject drugs [1]. While the overall number of new HIV infections in the United States remained stable between 2008 and 2010, new infections among MSM increased 12%, from 26,700 in 2008 to 29,800 in 2010 [2]. Among MSM, blacks and African Americans (hereafter referred to as blacks) are disproportionately affected by HIV. In 2010, black MSM accounted for 42% of all estimated incident HIV infections attributed to male-to-male sexual contact [2]. Black HIV-positive MSM are less likely to be on treatment compared to white MSM [3, 4], and such disparities in care and treatment among blacks may contribute to higher HIV incidence rates [5].

Efforts to reduce HIV-related health disparities include improving access to care services and increasing the proportion of MSM on antiretrovirals (ARV). Antiretrovirals lower HIV viral load, improve health outcomes, and reduce the likelihood of HIV transmission [6]. Timely linkage to care is a key step for initiation of ARV. In 2010, the White House released the National HIV/AIDS Strategy (NHAS), a comprehensive plan with measurable HIV targets to be achieved by 2015 [7]. These targets include increasing the proportion of newly diagnosed persons linked to clinical care within three months of HIV diagnosis from 65% to 85%. The NHAS also calls to increase the proportions of both HIV-diagnosed MSM and blacks with undetectable viral load by 20%. In 2013, the HIV Care Continuum Initiative was established by Presidential Executive Order to further focus implementation of the NHAS on activities to address gaps along steps of the HIV continuum of care, including early linkage to care and ARV treatment (Executive Order No. 13,649, 2013) [8].

Recommendations for initiating ARV in treatment-naïve persons have broadened over time. While recommendations were initially restricted to persons with AIDS-defining illness or a CD4 count less than 350 cells/mm^3^, guidelines were revised in 2009 to include those with a CD4 count less than 500 cells/mm^3^ [9]. With additional data supporting improved outcomes with early initiation of treatment [10, 11], ARV initiation was recommended for all HIV-infected individuals in 2012 [9].

CDC’s National HIV Behavioral Surveillance System (NHBS) monitors HIV-associated behaviors, including early linkage to care and ARV treatment, in MSM and other populations at high risk of HIV infection [12]. In order to monitor progress toward the NHAS targets and expanded treatment guidelines, we analyzed data from HIV-positive MSM from two cycles of NHBS (2008 and 2011), to determine if there was a difference in prevalence of 1) early linkage to care and 2) current ARV treatment between 2008 and 2011. We also examined differences between the two time points in early linkage to care and ARV treatment by demographic characteristics to quantify gaps and highlight progress in these two care measures.

## Materials and Methods

NHBS monitors HIV-associated behaviors and HIV prevalence in 20 cities with high AIDS burden among three populations at high risk for infection: MSM, injection drug users, and heterosexual adults at increased risk for HIV infection [12]. Cross-sectional behavioral data reported in this analysis are from MSM recruited for interviews and HIV testing through venue-based, time-space sampling (VBS) in NHBS surveys in 2008 and 2011. NHBS VBS procedures have been previously published [12, 13] and are briefly summarized here. First, staff identified venues frequented by MSM (e.g., bars, dance clubs, gyms, restaurants, parks, street locations, and social organizations) and days and times when men frequented those venues. In 2008, venues in which 75% of men attending were MSM were eligible for inclusion, while in 2011, this threshold was decreased to 50% due to more diverse venue attendee populations to produce a comparable number of eligible venues in 2011. A comparison of the response rates for the two years has been previously published [14]. Staff of funded sites determined venue eligibility through secondary data review, interviews, focus groups, or observations. Second, venues and corresponding day-time periods were selected randomly for recruitment events. Third, staff members systematically approached men at recruitment events to screen for eligibility (aged ≥ 18 years, lived in a participating city, and able to complete the interview in English or Spanish). In 2011, only men who reported ever having sex with another man were eligible. Although eligibility criteria differed, the same analysis criteria were used for both years in this analysis. Interviews were conducted by trained interviewers using a standardized questionnaire covering demographics, HIV-associated behaviors, and use of HIV prevention and testing services.

The two main outcomes in this analysis were early linkage to care and current ARV treatment. Early linkage to care was defined as a reported clinic visit for HIV care within three months of HIV diagnosis. HIV diagnosis date was ascertained from the questions “Before your test in __/__ [most recent test], did you ever test positive for HIV?” and, if yes, “When did you first test positive?” Early linkage to care was ascertained from the questions “Have you ever been seen by a doctor, nurse, or other health care provider for a medical evaluation or care related to your HIV infection?” and, if yes, “When did you first go to your health care provider after learning you had HIV?” Analyses for early linkage to care were restricted to MSM diagnosed with HIV three or more months prior to NHBS interview. Early linkage to care analyses were further restricted to those diagnosed with HIV in the three years prior to the study year (2006–2008 for the 2008 study year and 2009–2011 for the 2011 study year). Since only individuals diagnosed after 2008 can contribute to a difference in early linkage to care between 2008 and 2011, data from both 2008 and 2011 were restricted to the three years prior to the study year to create comparable samples. Current ARV treatment was defined as self-reported use at the time of NHBS interview, as ascertained by the question “Are you currently taking antiretroviral medicines to treat your HIV infection?” Self-reported HIV-positive MSM (defined as ever had sex with a man) with complete and valid interview data were included in analysis. Validity was assessed by the interviewer’s confidence in the respondent’s answers; interviewers received in-person training on administering the questionnaire and interviews they marked invalid were excluded from analysis. Confirmatory test results were not available for 225 of 1,144 self-reported HIV-positive men in 2008 and 285 of 1,337 men in 2011 who declined HIV testing; thus, self-reported HIV-positive status was used. A sensitivity analysis that excluded men without a positive confirmatory test did not change study results. Data for 2008 were restricted to men who reported ever having sex with another man to match the 2011 eligibility criterion. Data came from the 20 cities that contributed NHBS data in both 2008 and 2011: Atlanta, GA; Baltimore, MD; Boston, MA; Chicago, IL; Dallas, TX; Denver, CO; Detroit, MI; Houston, TX; Los Angeles, CA; Miami, FL; New Orleans, LA; Nassau-Suffolk, NY; Newark, NJ; New York, NY; Philadelphia, PA; San Diego, CA; San Francisco, CA; San Juan, PR; Seattle, WA; and Washington, DC.

Unadjusted prevalence ratios comparing the outcomes in 2011 to 2008 were calculated to explore differences over time by demographic characteristics (race, age, education, income, and insurance). Because the goal of this analysis was to describe early linkage to care and ARV treatment and how prevalence of these outcomes may have differed over time among demographic groups, behavioral and other risk factors were not considered. Adjusted prevalence ratios were calculated from linear mixed models with a Poisson distribution and log link. Each model included demographic characteristics and their interactions with year as fixed effects. City was included as a random effect to address correlated data within cities. The type of venue where recruitment occurred (bar, dance club, or other) was also included in models as a fixed effect to further account for some of the methodological complexities associated with VBS. Analysis of racial disparities in prevalence of ARV treatment adjusted for other demographic characteristics was conducted separately for 2008 and 2011 data and using combined 2008 and 2011 data. Model-adjusted prevalence of ARV treatment by racial category was estimated using ESTIMATE statements in SAS. All analyses were conducted using SAS 9.3 (SAS Institute Inc., Cary, NC, USA).

Activities for NHBS were approved by local institutional review boards (IRB) for each of the 20 participating cities. NHBS activities were determined to be research in which the Centers for Disease Control and Prevention was not directly engaged and, therefore, did not require review by CDC IRB. All participants were explicitly assured during the recruitment process of the anonymous nature of the survey and the HIV testing. No personal identifiers were collected during enrollment, interview, or testing. All participants provided oral informed consent to take part in the interview and to be tested for HIV. Oral consent was documented electronically on the survey instrument by interviewers for all participants and on hard copy as required by local IRBs. Because data collection was anonymous, written consent was not possible and participant names or other personal identifiers were not linked to any NHBS instruments. All consent procedures, including verbal consent, were approved by local IRBs (Table 1).

**Table 1:** Approving Institutional Review Boards (IRBs) for NHBS-MSM Activities (2008 and 2011).

City	Approving IRB
Atlanta	Georgia Department of Human Resources IRB (2008 only)
	Emory University IRB (2008 only)
	Georgia Department of Public Health (2011 only)
	Georgia Department of Community Health (2011 only)
Baltimore	Maryland Department of Health and Mental Hygiene IRB
	Johns Hopkins Bloomberg School of Public Health IRB
Boston	Massachusetts Department of Public Health Human Research Review Committee
	Boston University Medical Center IRB (2011 only)
Chicago	Chicago Department of Public Health IRB
Dallas	Texas Department of State Health Services IRB
	Texas A&M University Office of Research Compliance
Denver	Colorado Multiple IRB
Detroit	Michigan Department of Community Health IRB
Houston	Univ. of Texas Health Science Center Committee for the Protection of Human Subjects
Los Angeles	County of Los Angeles Public Health IRB
Miami	Florida Department of Health IRB
	University of Miami IRB (2008 only)
Nassau	State of New York Department of Health IRB
New Orleans	State of Louisiana Department of Health and Hospitals IRB
	Louisiana State University Health and Sciences Center IRB
New York City	New York City Department of Health and Mental Hygiene IRB
	National Development and Research Institute IRB (2008 only)
	John Jay College of Criminal Justice IRB (2011 only)
Newark	State of New Jersey Department of Health and Senior Services IRB
Philadelphia	City of Philadelphia Department of Public Health IRB
San Francisco	Univ. of California, San Francisco Committee on Human Research
San Juan	Univ. of Puerto Rico, Medical Sciences Campus IRB
San Diego	California Health and Human Services Agency Committee for the Protection of Human Subjects
Seattle	State of Washington Department of Social and Health Services IRB
Washington, DC	Government of the District of Columbia Department of Health IRB
	George Washington University Medical Center IRB

## Results

Data on NHBS recruitment in 2008 and 2011 are presented elsewhere [14]. Of 9,463 MSM who consented and had valid and complete interviews in 2008, 1,144 (12.1%) reported being HIV-positive. Of 9,819 MSM who consented and had valid and complete interviews in 2011, 1,337 (13.6%) reported being HIV-positive. Men included in the analysis sample differed by race between 2008 and 2011 (Table 2). While the proportion that was white decreased 8% between 2008 and 2011, the proportion that was black increased 7%. Men included in the 2011 sample were slightly younger than those in the 2008 sample and were more likely to have been diagnosed with HIV at a younger age. Education, income, and health insurance status were similar between the two years. More than half of men in 2008 were recruited at bars (52%), with fewer men recruited at other venues (33%) and dance clubs (15%). In 2011, the proportion of men recruited at bars and other venues was comparable (42% vs. 41%), with 17% recruited at dance clubs.

**Table 2:** Characteristics of HIV-positive men who have sex with men–NHBS, 2008 and 2011.

Characteristic	2008		2011	
	n	(%)	n	(%)
**Total**	1,144		1,338	
**Race/Ethnicity**				
Black	292	(25.5)	446	(33.5)
Hispanic/Latino	243	(21.2)	278	(20.9)
White	543	(47.5)	528	(39.6)
Other^b^	66	(5.8)	80	(6.0)
**Current age (years)**				
18–29	205	(17.9)	325	(24.3)
30–39	327	(28.6)	328	(24.5)
40–49	405	(35.4)	442	(33.0)
≥50	207	(18.1)	243	(18.2)
**Education**				
Less than high school	88	(7.7)	86	(6.4)
High school diploma or equivalent	277	(24.2)	309	(23.1)
Some college	414	(36.2)	504	(37.7)
College or higher	365	(36.5)	438	(32.8)
**Annual household income**				
0 to $19,999	439	(38.6)	525	(39.7)
$20,000 to $39,999	262	(23.0)	344	(26.0)
$40,000 to $74,999	236	(20.7)	270	(20.4)
$75,000 or more	201	(17.7)	183	(13.9)
**Current health insurance**				
No	282	(24.7)	277	(20.7)
Yes	862	(75.4)	1,060	(79.3)
**Years since HIV diagnosis**				
≤ 5 years from interview date	476	(42.4)	574	(43.5)
6–10 years from interview date	201	(17.9)	248	(18.8)
> 10 years from interview date	447	(39.8)	499	(37.8)
**Age at HIV diagnosis (years)**				
≤ 24	265	(23.6)	418	(31.6)
25–29	274	(24.4)	295	(22.3)
30–39	410	(36.5)	414	(31.3)
≥ 40	175	(15.6)	194	(14.7)
**Venue type** ^**c**^				
Bar	596	(52.1)	565	(42.2)
Dance club	172	(15.0)	222	(16.6)
Other^d^	376	(32.9)	551	(41.2)
**City**				
Atlanta, Georgia	14	(1.2)	100	(7.4)
Baltimore, Maryland	52	(4.6)	60	(4.5)
Boston, Massachusetts	32	(2.7)	44	(3.3)
Chicago, Illinois	59	(5.2)	83	(6.2)
Dallas, Texas	73	(6.5)	105	(7.9)
Denver, Colorado	106	(9.3)	80	(6.0)
Detroit, Michigan	38	(3.3)	55	(4.1)
Houston, Texas	98	(8.6)	73	(5.5)
Los Angeles, California	71	(6.2)	77	(5.8)
Miami, Florida	77	(6.7)	84	(6.3)
Nassau-Suffolk, New York	20	(1.8)	13	(1.0)
Newark, New Jersey	73	(6.5)	66	(4.9)
New Orleans, Louisiana	12	(1.1)	39	(2.9)
New York, New York	93	(8.1)	76	(5.7)
Philadelphia, Pennsylvania	33	(2.9)	50	(3.7)
San Diego, California	77	(6.7)	74	(5.5)
San Francisco, California	96	(8.4)	110	(8.2)
San Juan, Puerto Rico	14	(1.2)	10	(0.8)
Seattle, Washington	52	(4.6)	72	(5.4)
Washington, D.C.	55	(4.8)	67	(5.0)

Numbers may not add to total due to missing values.

^a^Hispanic/Latinos can be of any race

^b^Includes MSM reporting American Indian or Alaska Native, Asian, Native Hawaiian or Pacific Islander, other race, or multiple races

^c^ Venue type refers to the type of venue where the participant was recruited

^d^Other venues include gyms, restaurants, parks, street locations, social organizations, and other places where MSM congregate

For the early linkage to care analysis, 52 MSM in 2008 and 57 men in 2011 were diagnosed with HIV in the three months prior to the NHBS interview and were excluded. An additional 20 MSM in 2008 and 17 MSM in 2011 were excluded because they were diagnosed in the same year as the interview and had an unknown month of diagnosis. Two men in 2008 were missing data on first visit for HIV care. After excluding 833 men diagnosed before 2006 in the 2008 sample and 968 men diagnosed before 2008 in the 2011 sample, 236 men in 2008 and 291 men in 2011 were included in analyses (Table 3). In both years, early linkage to care was more likely among those with higher education and income and with current insurance. Overall, prevalence of early linkage to care did not differ over time (79% in 2008 and 83% in 2011, *P* = 0.44). In addition, early linkage to care did not differ among demographic sub-groups between 2008 and 2011. Early linkage to care was reported by a higher proportion of black MSM in 2011 (83%) than 2008 (72%), but this increase was not statistically significant (*P* = 0.11). Multivariable analyses were not done due to the lack of statistical significance of bivariate analyses.

**Table 3:** Prevalence of early linkage to HIV care and prevalence ratios comparing prevalence of early linkage to care^a^ in 2011 to 2008 among men who have sex with men–NHBS.

Characteristic	2008			2011				
	No. in sample	n (%)		No. in sample	n (%)		Unadjusted^b^ PR (95% CI)	*P*-value
**Overall**	236	187	(79.2)	291	241	(82.8)	1.05 (0.93–1.18)	.44
**Race/Ethnicity**								
Black	72	52	(72.2)	119	99	(83.2)	1.15 (0.96–1.38)	.11
Hispanic/Latino^c^	53	42	(79.3)	61	54	(88.5)	1.12 (0.96–1.30)	.15
White	90	77	(85.6)	91	74	(81.3)	0.90 (0.55–1.48)	.66
Other^d^	21	16	(76.2)	19	13	(68.4)	0.95 (0.81–1.11)	.51
**Age at HIV diagnosis (years)**								
≤ 24	61	48	(78.7)	116	95	(81.9)	1.04 (0.85–1.27)	.69
25–29	50	37	(74.0)	52	43	(82.7)	1.12 (0.89–1.40)	.32
30–39	74	56	(75.7)	72	62	(86.1)	1.14 (0.91–1.43)	.26
≥ 40	51	46	(90.2)	51	41	(80.4)	0.89 (0.74–1.07)	.20
**Education**								
High school or less	85	66	(77.7)	105	81	(77.1)	0.99 (0.86–1.15)	.93
More than high school	151	121	(80.1)	186	160	(86.0)	1.07 (0.94–1.23)	.28
**Annual household income**								
$0 to $19,999	92	70	(76.1)	147	119	(81.0)	1.06 (0.92–1.23)	.37
≥$20,000	143	117	(81.1)	142	120	(84.5)	1.03 (0.90–1.19)	.63
**Current insurance**								
No	85	64	(75.3)	86	62	(72.1)	0.96 (0.77–1.19)	.67
Yes	151	123	(81.5)	205	179	(87.3)	1.07 (0.95–1.21)	.23

Abbreviations: CI, confidence interval; MSM, men who have sex with men; PR, prevalence ratio

^a^Defined as a self-reported clinic visit for HIV care within three months of HIV diagnosis, restricted to those diagnosed with HIV in the three years prior to the study year (2006–2008 for the 2008 study year and 2009–2011 for the 2011 study year)

^b^Reference is 2008; PR corresponds to the interaction term with year; city is included as a random effect

^c^Hispanic/Latinos can be of any race

^d^Includes MSM reporting American Indian or Alaska Native, Asian, Native Hawaiian or Pacific Islander, other race, or multiple races

**Fig 1:**
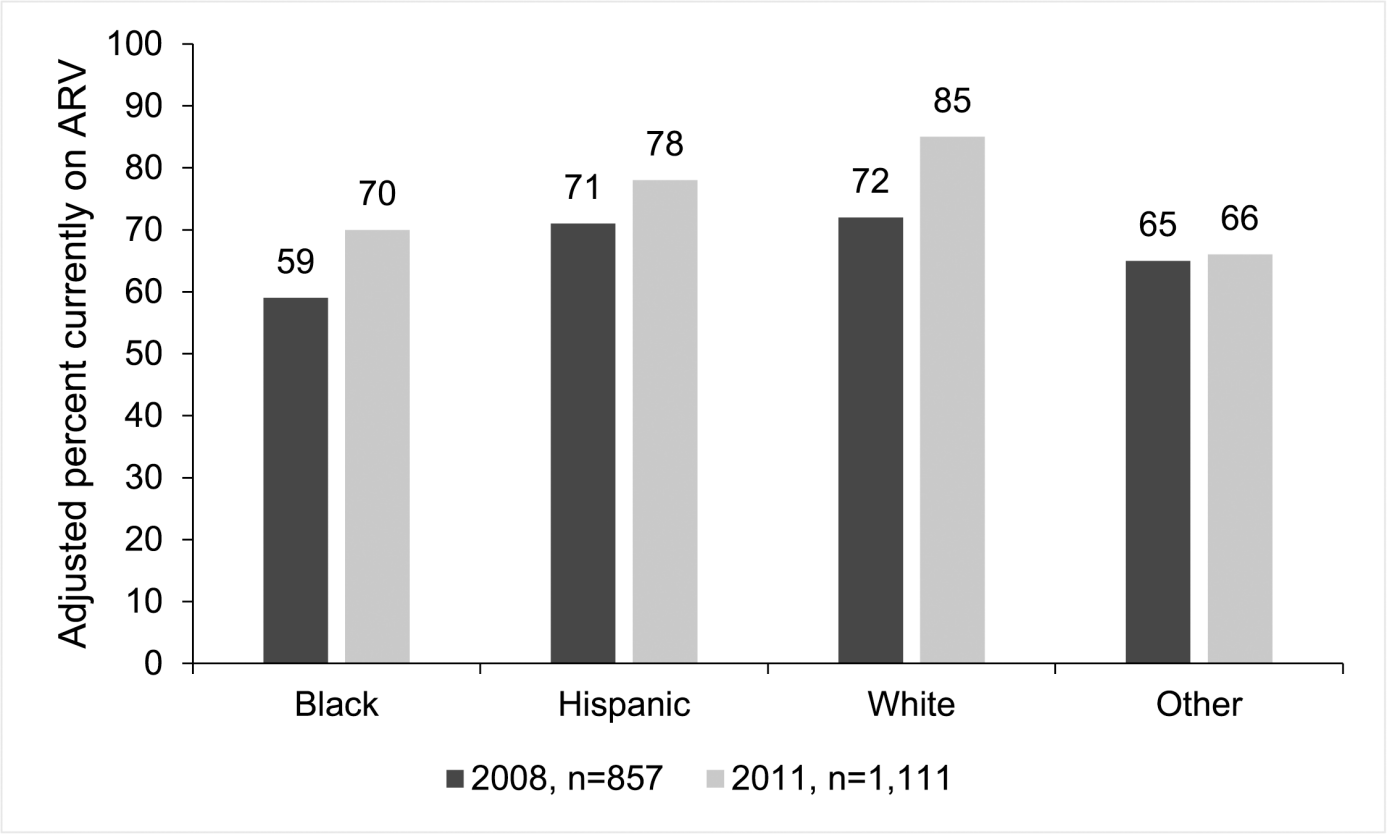
Adjusted prevalence^a^ of current ARV treatment by race/ethnicity among MSM—NHBS, 2008 and 2011. Adjusted prevalences from a model adjusted for year, current age, annual household income, current insurance, venue type where recruitment occurred, and city (random effect) show that the percent of blacks currently on antiretroviral therapy is significantly less than the percent of whites currently on antiretroviral therapy in both years. ^**a**^Adjusted prevalence estimated from the following model: current ARV = α + β1*race + β 2*age + β 3*current insurance + β 4*income + β 5*venue type + β 6*year + β 7*race*year + β 8*age*year + β 9*current insurance*year + β 10*income*year; city is included as a random effect; adjusted prevalence ratio based on combined 2008, 2011 data comparing whites to blacks was 1.09 (CI: 1.02–1.16); ^**b**^Hispanics can be of any race; ^**c**^Includes MSM reporting American Indian or Alaska Native, Asian, Native Hawaiian or Pacific Islander, other race, or multiple races.

For the current ARV treatment analysis, all self-reported HIV-positive MSM interviewed in 2008 (n = 1,144) and 2011 (n = 1,337) were eligible for inclusion. Two MSM in 2008 and one MSM in 2011 were missing data on current ARV treatment, leaving 1,142 in 2008 and 1,336 in 2011 for the analysis (Table 4). In both years, a higher percentage of ARV treatment was observed among whites, older age groups, MSM with higher education and income, and those with health insurance. Overall, prevalence of ARV treatment increased from 69% in 2008 to 79% in 2011 (*P*<0.0001). ARV treatment also increased among most race/ethnicity groups, younger age groups (<40 years), and MSM with higher education. Increases in ARV treatment were seen across income and insurance groups. There was a significant racial disparity between white and black MSM in current ARV treatment in both years (Fig 1). Whites were 19% more likely than blacks to report current ARV treatment when data for 2008 and 2011 were combined (*P*<0.001). In the multivariable analysis, whites were 9% more likely than blacks to report current ARV treatment (*P* = 0.01). Separate analysis of 2008 and 2011 data produced similar results (data not shown).

**Table 4:** Prevalence of current ARV treatment and prevalence ratios comparing prevalence of current ARV treatment in 2011 to 2008 among MSM–NHBS.

Characteristic	2008			2011						
	No. in sample	n (%)		No. in sample	n (%)		Unadjusted^a^ PR (95% CI)	*P*-value	Adjusted^b^ PR (95% CI)	*P*-value
**Overall**	1,142	790	(69.2)	1,336	1,049	(78.5)	1.14 (1.08–1.19)	< .0001	1.20 (1.11–1.31)	.0003
**Race/Ethnicity**										
Black	291	177	(60.8)	444	315	(71.0)	1.17 (1.04–1.31)	.01	1.25 (1.09–1.42)	.001
Hispanic/Latino^c^	243	170	(70.0)	278	220	(79.1)	1.13 (1.00–1.27)	.04	1.22 (1.08–1.39)	.002
White	542	400	(73.8)	528	453	(85.8)	1.16 (1.10–1.23)	.44	1.29 (1.16–1.44)	< .0001
Other^d^	66	43	(65.2)	80	56	(70.0)	1.07 (0.89–1.29)	< .0001	1.05 (0.86–1.29)	.63
**Current age (years)**										
18–29	205	78	(38.1)	323	185	(57.3)	1.51 (1.22–1.86)	.0003	1.46 (1.22–1.76)	.0001
30–39	325	205	(63.1)	328	258	(78.7)	1.25 (1.11–1.40)	.0003	1.24 (1.07–1.42)	.004
40–49	405	334	(82.5)	442	386	(87.3)	1.06 (0.99–1.13)	.08	1.06 (0.95–1.19)	.29
≥ 50	207	173	(83.6)	243	220	(90.5)	1.08 (1.00–1.18)	.06	1.08 (0.96–1.21)	.19
**Education**										
High school or less	364	238	(65.4)	395	285	(72.2)	1.10 (0.99–1.23)	.07	—	—
More than high school	778	552	(71.0)	940	763	(81.2)	1.14 (1.07–1.22)	.0006	—	—
**Annual household income**										
$0 to $19,999	437	290	(66.4)	524	384	(73.3)	1.10 (1.01–1.21)	.04	1.18 (1.06–1.31)	.005
≥$20,000	699	495	(70.8)	796	652	(81.9)	1.16 (1.10–1.22)	< .0001	1.22 (1.12–1.34)	.0002
**Current insurance**										
No	281	142	(50.5)	277	167	(60.3)	1.19 (1.00–1.43)	.05	1.23 (1.06–1.42)	.008
Yes	861	648	(75.3)	1,058	881	(83.3)	1.11 (1.07–1.15)	< .0001	1.17 (1.10–1.24)	< .0001

Abbreviations: ARV, antiretroviral; CI, confidence interval; MSM, men who have sex with men; PR, prevalence ratio

^a^Reference is 2008; PR corresponds to the interaction term with year

^b^Reference is 2008; PR corresponds to the interaction term with year; model includes year, race/ethnicity, current age, annual household income, current insurance, venue type where recruitment occurred (bar, dance club, or other), and their interactions with year as fixed effects; city is included as a random effect

^c^Hispanic/Latinos can be of any race

^d^Includes MSM reporting American Indian or Alaska Native, Asian, Native Hawaiian or Pacific Islander, other race, or multiple races.

Data on ARV use in the continuum of care are often presented among those retained in HIV care (the previous step in the continuum). While we did not make this restriction in our analysis, we present these data in order to compare our ARV use prevalence to other studies. When the sample of MSM was restricted to those who reported being retained in care (defined as an MD visit for HIV in the 6 months prior to interview, n = 905 in 2008 and n = 1,101 in 2011), the prevalence of ARV use was 79% in 2008 and 87% in 2011 (*P*<0.0001) (data not shown), a similar increase to the unrestricted analysis.

## Discussion

While the prevalence of early linkage to care among MSM did not increase significantly between 2008 and 2011, the prevalence of ARV treatment increased 10% overall and among most demographic groups. These data suggest that those who have linked to care are increasingly initiating ARV, in accordance with the more inclusive guidelines introduced in 2009.

There is a large disparity between black and white MSM with respect to current ARV treatment despite similar prevalence for early linkage to care between the two groups. Studies have demonstrated that black HIV-positive individuals are less likely than whites to be prescribed ARV and to adhere to ARV regimens once prescribed [15–18]. The adjusted 9% difference in prevalence of ARV treatment between black and white MSM in the NHBS sample is similar to a 7% difference (adjusted for age, lapse in health coverage, poverty, time since diagnosis, and disease stage) described among black and white MSM attending 461 US care facilities in the Medical Monitoring Project [19]. These disparities are much lower than those described in a meta-analysis of three quantitative studies of MSM done prior to 2003 that stratified ARV treatment by race [4]. That analysis found that HIV-positive black MSM were 57% less likely to report taking ARV compared to white MSM (OR: 0.43, 95% CI: 0.30–0.61), although the contributing studies were small or nonrepresentative and were not adjusted for age and other covariates. While the disparity among MSM is smaller in NHBS, it is worth noting that it did not improve between the 2008 and 2011 data collection cycles.

Our data also show a disparity between younger and older MSM. Younger MSM were much less likely to be on ARVs compared to older MSM (57% among those < 30 years vs. 86% among those ≥ 30 years in 2011). Maximizing care and treatment for young MSM is important as HIV case surveillance data show that the only age group among MSM for which new HIV infections increased from 2008 to 2010 was MSM aged 13 to 24 years [2]. While racial disparities in HIV prevalence and treatment have been widely described since the early years of the epidemic [20, 21], recent data show alarming rates of HIV infection among young MSM, especially young blacks [22].

The prevalence of early linkage to care among this sample of MSM was relatively high, at 83% in 2011. This estimate is similar to an analysis of early linkage to care among MSM in the U.S. for 2009, which estimated that 80% of MSM were linked to care within three months of diagnosis [5]. Both analyses suggest that the NHAS goal of 85% linked to care early by 2015 is feasible among HIV-positive MSM. Estimates of ARV use among NHBS MSM retained in care are also comparable to Hall et al (87% among NHBS MSM compared to 90% among MSM in Hall et al) [5]. Our estimate may be slightly lower due to a higher prevalence of young MSM in our sample, who were less likely to be on ARVs.

Ongoing changes in the U.S. health-care system offer opportunities to improve use of clinical preventive services by MSM. The Patient Protection and Affordable Care Act of 2010 (as amended by the Healthcare and Education Reconciliation Act of 2010 and referred to collectively as the Affordable Care Act [ACA]) expands insurance coverage, consumer protections, and access to primary care and emphasizes prevention in addition to care and treatment (see http://aids.gov/federal-resources/policies/health-care-reform/). Under the ACA, most new health insurance plans must cover HIV testing for everyone aged 15 to 65 without additional cost-sharing, such as copays or deductibles (payments made by the beneficiary). In addition, many HIV-infected MSM are newly eligible for Medicaid coverage and others are eligible to purchase private insurance through the marketplaces (websites where individuals can compare insurance plans and enroll in coverage) [23]. However, significant barriers to care continue, as HIV-infected MSM will remain uninsured if they reside in a state that is not expanding Medicaid [24]. Many of the states that did not expand Medicaid are located in the southern U.S., where prevalence of HIV is higher. In addition, subsidies for private coverage are not available to people with incomes below 100 percent of poverty, so many HIV-infected MSM may not qualify for these subsidies [22].

Our analyses are subject to several limitations. First, NHBS is not a nationally representative sample, so results may not be generalizable to all cities or to all MSM in participating cities. Our population of inference is limited to MSM who reside in and attend MSM venues in the NHBS cities. Second, our data are collected through face-to-face interviews and our measures of early linkage to care and ARV treatment are based on self-reported data and might be subject to social desirability and recall bias. Social desirability would lead to overestimation of prevalence estimates, while the direction of bias due to recall error is unknown. However, our results are based on differences between 2008 and 2011 and are less likely to be affected by these biases than the point estimates themselves provided the biases remained consistent over time. The data are also subject to variability related to interviewer reliability and skill. There were differences in eligibility in 2008 and 2011 but the same analysis criteria were used for both years. The NHBS questionnaire has undergone minor updates between MSM cycles, but the items used to determine early linkage to care and ARV use were identical in 2008 and 2011. In addition, this is a cross-sectional analysis that does not permit inference regarding causation. The analysis is limited to two time points and cannot be interpreted as a trend nor as resulting from changes to policy or practices that occurred between these time points. Our analysis also did not include data on ARV adherence or viral load suppression, so we cannot say whether MSM currently on ARV are at reduced risk of HIV transmission. We therefore do not have viral suppression data to compare to the NHAS goals for MSM overall and among black MSM. Finally, data are not weighted to account for the complex sampling methodology used to recruit MSM. Point estimates may therefore be biased by over- or under-represented subgroups of the sample. Therefore, the point estimates should be interpreted with caution, especially those for the total sample. However, multivariate analysis of differences across years should not be affected by a lack of weighting, especially given the consistency of data distribution between 2008 and 2011 [25].

In summary, our analysis demonstrated a significant increase in ARV treatment between 2008 and 2011 among HIV-positive MSM, encompassing a time period when the National HIV/AIDS Strategy was introduced and treatment guidelines were expanded. While there was not an increase in early linkage to care between 2008 and 2011, the high prevalence of early linkage to care is encouraging, especially with the increasing evidence supporting treatment as prevention and expanded treatment guidelines released in 2012. Efforts are needed, however, to decrease barriers to ARV provision and adherence among black MSM in order to reduce the present disparity in treatment.
